# Parents’ perceptions of their child’s weight among children in their first year of primary school: a mixed-methods analysis of an Australian cross-sectional (complete enumeration) study

**DOI:** 10.1038/s41366-022-01068-5

**Published:** 2022-01-24

**Authors:** Kathleen O’Brien, Jason Agostino, Karen Ciszek, Kirsty A. Douglas

**Affiliations:** 1 Academic Unit of General Practice, Australian National University, Canberra, ACT Australia; 2 Academic Unit of General Practice, ACT Health, Canberra, ACT Australia; 3 Academic Unit of General Practice, ACT Health / Australian National University, Canberra, ACT Australia

**Keywords:** Paediatrics, Public health

## Abstract

**Background/Objectives:**

To describe trends in overweight/obesity in early childhood for all children and those whose parents are concerned about their weight. To describe parents’ perceptions of their child’s weight and differences by their child’s anthropometric and sociodemographic factors.

**Subjects/Methods:**

Analysis of the Kindergarten Health Check, a survey of all children enrolled in their first year of primary education in the Australian Capital Territory. Analysis of detailed data for 2014–2017, including qualitative analysis of parents’ comments on weight, and trends for 2001–2017.

**Results:**

71,963 children participated in the survey between 2001 and 2017 (20,427 between 2014 and 2017). The average age of children (2001–2017) was 5 years and 9.6 months at the time of their physical health check. 2377 children (3.5%) were classified as obese based on measured body mass index (BMI) between 2001 and 2017, and a further 7766 (11.6%) overweight. Similar proportions were seen for 2014–2017. Among children with overweight/obesity in 2014–2017, 86.4% of parents (2479/2868) described their children’s weight as healthy and 13.3% (382/2868) as overweight/obese. Just 11.5% (339/2946) of parents whose children were later measured with overweight/obesity identified having a concern about their child’s weight.

Parental comments varied widely and were often incongruent with the known health risks associated with their child’s measured BMI. Comments from parents whose children were measured as obese often were normalising e.g., “*born big, always big. Definitely NOT overweight, just bigger all over”*, whilst parents of children in the healthy range expressed concerns about underweight.

**Conclusion:**

Parents do not accurately perceive their child’s weight and few document concerns, even among children measuring in the obese BMI category. This lack of concern makes early interventions challenging as parents are in the “pre-contemplative” stage of behaviour change and may see public health campaigns or clinicians’ attempts to address their child’s weight as irrelevant or unhelpful.

## Introduction

Overweight and obesity is a problem in Australia and worldwide, for children and adults, and the prevalence is increasing [1–3]. One in five (20.2%) Australian children aged 5–7 years were classified as overweight or obese in 2007–08 compared to 24.1% in 2011–12, increasing to over one in four (27.5%) in 2017–18 [4–6].

Children with overweight are at increased risk of both physiological and psychological difficulties [3]. A systematic review of obesity in Australian children found children and adolescents with obesity to be at increased risk of multiple physical and psychological comorbidities compared to healthy-weight children [7]. Overweight in childhood is associated with higher risk of being overweight as an adult, which is associated with higher rates of metabolic diseases, cardiovascular diseases and some cancers [3].

The causes of childhood obesity are complex, and include the interplay of environmental factors with maternal health, genetics and epigenetics, physiological factors and some medications [3, 8–10]. Genetic and physiological factors are not generally considered sufficient to explain the rapid increase in childhood obesity rates, with growing up in so-called obesogenic environments—those that promote weight gain—a key factor [3, 8, 9]. Parents/guardians/carers play an important part in helping children navigate these environments and in modelling healthy choices [11, 12]. In their recommendations to help combat child obesity, the World Health Organization advises providing guidance to parents and others on what constitutes healthy body weight and associated behaviours such as physical activity and good nutrition [3]. Children’s lifestyle is influenced by their families, and parents who are concerned about their child’s weight are more likely to employ strategies to improve the family diet, increase their child’s exercise and limit their screen time [11, 13]. However in their meta-analysis Lundahl et al. [14] found around half of parents underestimated their child’s weight in children with overweight or obesity, and one in seven underestimated weight in healthy weight children [14]. Accurately perceiving a child’s weight enables a parent to identify when changes may be required in the management of their child’s health.

Early intervention is well-known to be effective and important in reversing and mitigating the long term effects of childhood obesity [15]. Despite the complex aetiology of obesity, identifying characteristics associated with parental concern (or lack thereof) about their child’s weight, and accuracy or otherwise of how they perceive their child’s health, may help identify those children who would most benefit from additional support. Furthermore, exploring parents’ perceptions of their child’s weight in context of comments they have made may help us understand where perception differs from measured assessment in order to provide education and support.

The aims of our study were to:

- describe prevalence of overweight and obesity in early childhood and explore changes over time,
- explore parents’ perceptions of and concerns about their child’s weight by the child’s anthropometric and sociodemographic factors,
- explore factors associated with parents’ perception of their child’s weight and why that may differ from their child’s measured body mass index (BMI),
- explore comments made by parents on their child’s weight in order to understand why their perceptions may not align with their child’s anthropometric measurements.

## Methods

The Kindergarten Health Check (KHC) is an annual cross-sectional complete enumeration survey of all children in the Australian Capital Territory (ACT) in their first year of full-time primary education (Kindergarten). It consists of a questionnaire completed by parents at the start of the school year, and a physical health check performed by school health nurses later in the school year. Our study analyses trends for 2001–2017, with more detailed analysis of data limited to 2014–2017 due to changes in the survey instrument following a major review and refresh of all survey instruments by the KHC Governance Committee in 2013 [16]. Measured height and weight were collected for the entire period, while questions around parental perceptions and concerns were refined from 2014. The data collection and consent process has been granted ethics approval through the ACT Health Human Research Ethics Committee (ETHLR.13.316). Ethics approval for our study was granted by the ACT Health Human Research Ethics Committee’s Low Risk Sub-Committee (ETHLR.16.029 and 2019/LRE/0199).

The KHC questionnaire collects demographic information (e.g., parent-reported Indigenous identification, sex, date of birth) and information on selected conditions and behaviours. From 2014, parents/guardians were asked to indicate how they would describe their child’s weight (choosing from underweight, healthy weight, overweight, obese, or don’t know) and whether they had any concerns about height or weight, with an option for parents to provide free-text comments following this. The 2017 questionnaire has been included in Supplementary File 1.

Kindergarten starts on or about 1 February each year, and questionnaires are typically returned within the first month of school. In order to calculate the child’s age at the time of the questionnaire, these were assigned an average date of completion of 14 February of that school year. Average age at time of the physical health screen was taken as the difference between reported date of birth and date of assessment.

The physical health check includes measurement of height and weight by school health nurses.

Children are measured after removing their shoes, jumper and/or jacket, and hair ornaments.

Weight is measured to the nearest 0.1 kg on annually calibrated digital scales placed on a hard level surface. Height is measured to the nearest 0.1 cm using a stadiometer with attached headboard [17]. Body mass index was calculated and classified into weight categories based on extended international (IOTF) age- and sex-specific cut-offs [18]. Where numbers were small, the overweight and obese categories were combined, as were the underweight and healthy weight categories.

We linked the 2014–2017 KHC with an indicator of socioeconomic advantage—the 2016 Index of Relative Socio-Economic Disadvantage (IRSD), one of the Socio-Economic Indexes for Areas (SEIFA) produced by the Australian Bureau of Statistics (ABS)—at the finest geographic level available, the Statistical Area Level 1 (SA1) [19]. SA1 was geocoded from home address. We created quintiles of socioeconomic disadvantage for the ACT by ranking the IRSD scores for ACT SA1 regions and dividing them into five equal cohorts.

Our analysis comprised two arms—a quantitative analysis and qualitative analysis.

Throughout this paper, references to parents with respect to perceptions, concerns and comments, is taken to include caregivers such as guardians.

### Quantitative statistical analysis

Descriptive statistics (mean and standard deviation for continuous variables, and number and percentages for categorical variables) were produced to summarise the characteristics of the study population, for 2001–2017, and 2014–2017. The prevalence of overweight and obesity was shown by the number and percentage of children in these BMI categories, with changes over time described by mean BMI stratified by year and sex. Descriptive data exploring the relationship between weight, parents’ perceptions and concerns was presented as: number and percent of parents’ perception and number/percent indicating concerns each stratified by measured BMI category; BMI score plotted against perception, stratified by whether parents had indicated concerns.

Finally, to identify factors associated with measured BMI and parents’ perception of and concerns about their child’s weight, data were stratified into healthy weight/overweight or obese and comparative statistics undertaken. *T*-tests were conducted to compare mean BMI by presence of parental concerns, and parental perceptions, as BMI is normally distributed. Chi-square tests were used to compare categorical variables. For *t*-tests and chi-square tests, *p* < 0.05 was considered statistically significant.

Missing data were assumed to be random and so excluded from comparative analysis.

Analysis was conducted in SPSS version 25.

### Qualitative analysis

Parents’ free-text comments on their child’s height/weight were extracted from the database for 2014–2017, with associated information such as sex, parental perceptions, and derived BMI. These comments were provided within the initial questionnaire after parents selected whether they had any concerns about their child’s height or weight but before the child’s height and weight were measured by the school health team. During analysis the comments were stratified by the child’s calculated BMI category, and whether the parents had concerns about weight. Inductive thematic analysis was undertaken by two researchers (clinician researchers with experience in working with families and children—JA and KD) who individually coded and identified themes followed by joint review of emergent themes prior to final agreement [20].

## Results

### Quantitative analysis

There were 81,420 children enrolled in ACT Kindergartens between 2001 and 2017, of whom 71,963 (88.1%) participated in the KHC (Table 1 and Academic Unit of General Practice unpublished analysis of ACT Education Directorate school census data). 22,569 children were enrolled in Kindergarten in ACT schools during the period 2014–2017, of whom 20,427 (90.5%) participated in the KHC.

**Table 1:** Characteristics of children in the Kindergarten Health Check, 2001–2017.

Years		2014–2017		2001–2017	
Characteristic		Mean	(SD)	Mean	(SD)
Average age at completion of questionnaire^a^ (months)		63.7	3.83	63.9	8.64
Age at physical health screen (months)^b^		69.0	4.27	69.6	7.43
Average number of months between completion of questionnaire^a^ and physical health screen^b^		4.8	1.90	5.3	2.25
		Number	Percent	Number	Percent
Sex	Male	10,526	51.5	36,732	51.0
	Female	9901	48.5	35,231	49.0
	Total	20,427	100	71,963	100.0
Indigenous status	Aboriginal or Torres Strait Islander	491	2.4	1422	2.0
	Not Aboriginal or Torres Strait Islander	19,878	97.6	68,990	98.0
	Total	20,369	100	70,412	100.0
	Not stated	58	n.a.	1551	n.a.
Socioeconomic advantage	Most disadvantaged (Q1)	3615	18.4	n.a.	
	Quintile 2	3963	20.2	n.a.	
	Quintile 3	4310	21.9	n.a.	
	Quintile 4	3959	20.2	n.a.	
	Least disadvantaged (Q5)	3786	19.3	n.a.	
	Total	19,633	100.0	n.a.	
	Not available	794	n.a.	n.a.	
Body mass index category	Underweight	1162	6.1	4576	6.8
	Healthy weight	14,963	78.2	52,413	78.1
	Overweight	2322	12.1	7766	11.6
	Obese	693	3.6	2377	3.5
	Total	19,140	100.0	67,132	100.0
	Not available	1287	n.a.	4 831	n.a.
Parental perception of weight	Underweight	1265	6.5	n.a.	
	Healthy weight	17,790	91.3	n.a.	
	Overweight	416	2.1	n.a.	
	Obese	15	0.1	n.a.	
	Total	19,486	100	n.a.	
	Not stated	941	n.a.	n.a.	
Parental concern about weight	Yes	1421	7.1	4328	6.1
	No	18,553	92.9	67,181	93.9
	Total	19,974	100.0	71,509	100.0
	Not stated	453	n.a.	454	n.a.
Whether has a usual GP	Yes	17,434	86.6	62,583	88.3
	No	2696	13.4	8329	11.7
	Total	20,130	100.0	70,912	100.0
	Not stated	297	n.a.	1051	n.a.
All children		20,427	100.0	71,963	100.0

*n.a.* not available.

^a^14 February of the school year taken as the average date of completion.

^b^Excludes 1258 and 4625 children from 2014–2017 and 2001–2017 respectively, for whom age at physical health screen was not available.

Children in the 2014–2017 cohort were aged an average of 63.7 months (SD 3.83), or 5 years and 3.7 months, at completion of the questionnaire (Table 1). The average age of those who participated in the physical health screen was 69.0 months (SD 4.27), or 5 years and 9 months, leaving an average of 4.8 months (SD 1.90) between the reporting of parents’ perceptions and the time of the physical health screen (Table 1).

#### Prevalence of and trends in overweight and obesity

Over three-quarters (78.1%) of children had a BMI in the healthy range during the period 2001–2017 (Table 1). 11.6% were measured as overweight and a further 3.5% obese. Similar proportions were observed over 2014–2017.

**Fig. 1:**
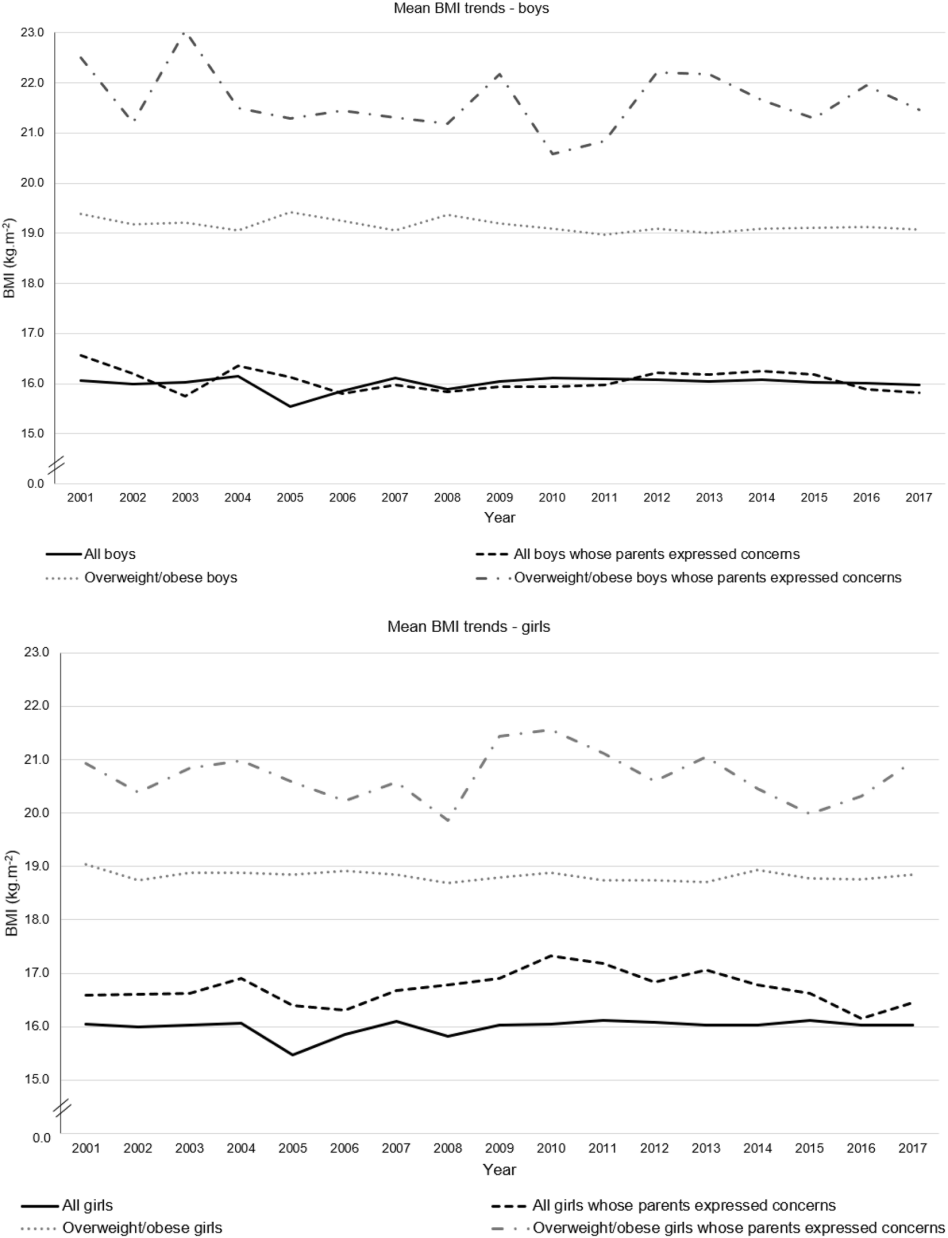
Trends in average BMI, by sex, 2001–2017. Trends are presented separately for all children and those whose parents expressed concerns, for all children and those who measured overweight/obese.

Both average weight and average height were relatively stable over the period 2001–2017, for boys and girls, with outliers for height seen in 2005. This appears to be due to systematic measurement error in 2005, which is explored further in the discussion. Excluding 2005 (due to concerns about measurement error), average height ranged from 1.16 m to 1.17 m for boys, and 1.14 m to 1.15 m for girls. Over this same period, average weight ranged from 21.5 kg to 22.2 kg for boys, and 21.0 kg to 21.6 kg for girls. Fig 1 shows trends in mean BMI for all children and those classified as overweight/obese for the period 2001–2017. Again excluding 2005, average BMI ranged from 15.86 kg.m^2^ to 16.16 kg.m^2^ for boys, and 15.82 kg.m^2^ to 16.12 kg.m^2^ for girls. For a boy of average height (1.17 m in our survey population) this is equivalent to a variation of 830 g; for a girl (average height of 1.15 m) this is equivalent to 865 g. Children in the overweight/obese BMI range whose parents expressed concerns about their weight, had higher BMI on average than all children.

#### Relationship between parents’ perceptions, concerns, and child’s characteristics

6.1% of parents indicated they had concerns about their child’s weight over the period 2001–2017 (7.1% in 2014–2017) (Table 1).

Among children who were measured as overweight or obese, 86.4% of parents described their children as being of healthy weight and only 13.3% accurately perceived them as overweight or obese. Only 5.6% of children with overweight were identified as such by their parents, and less than half (41.3%) of children with obesity were perceived as overweight or obese by their parents. Overall, there was concordance between measured BMI category and parental perception of weight category in 76.7% (14,021/18,285) of all children (Table 2). Only 2.1% (13/622) of children who were measured in the obese BMI category were considered to be so by their parents.

**Table 2:** Parents’ perception of their child’s weight by measured body mass index, 2014–2017.

Measured BMI	Parents’ perception of their child’s weight						Measured BMI percent of total
	Underweight	Healthy weight	Overweight	Obese	*Overweight or obese*	Total	
	**Number (per cent of measured BMI total)**^**a**^						**Percent**^**b**^
Underweight	364 (34.1)	700 (65.7)	2 (0.2)	0 (0.0)	*2 (0.2)*	1066	5.8
Healthy weight	811 (5.7)	13,519 (94.2)	20 (0.1)	1 (0.0)	*21 (0.1)*	14,351	78.5
Overweight	7 (0.3)	2114 (94.1)	125 (5.6)	0 (0.0)	*125 (5.6)*	2246	12.3
Obese	0 (0.0)	365 (58.7)	244 (39.2)	13 (2.1)	*257 (41.3)*	622	3.4
*Overweight or obese*	*7 (0.2)*	*2479 (86.4)*	*369 (12.9)*	*13 (0.5)*	*382 (13.3)*	*2868*	*15.7*
Total	1182 (6.5)	16,698 (91.3)	391 (2.1)	14 (0.1)	*405 (2.2)*	18,285	100.0

Cells highlighted in green indicate concordance between parental perception and measured BMI category.

^a^Row percentages.

^b^Column percentages.

Nearly 6% of children in the healthy weight range were described as underweight, and 65.7% of underweight children were described as healthy weight (Table 2).

#### Factors associated with parents’ perceptions of their child’s weight and their measured BMI

For children whose parents expressed concern about their weight, average BMI for boys was relatively stable, ranging from 15.76 kg.m^−2^ and 16.57 kg.m^−2^, and for girls from 16.15 kg.m^−2^ to 17.33 kg.m^−2^, without any clear overall trends seen (Fig. 1). This equates to a range of about 1.11 kg for a boy of average height (1.17 m in 2001–2017) and 1.56 kg for a girl of average height (1.15 m in 2001–2017).

**Fig. 2:**
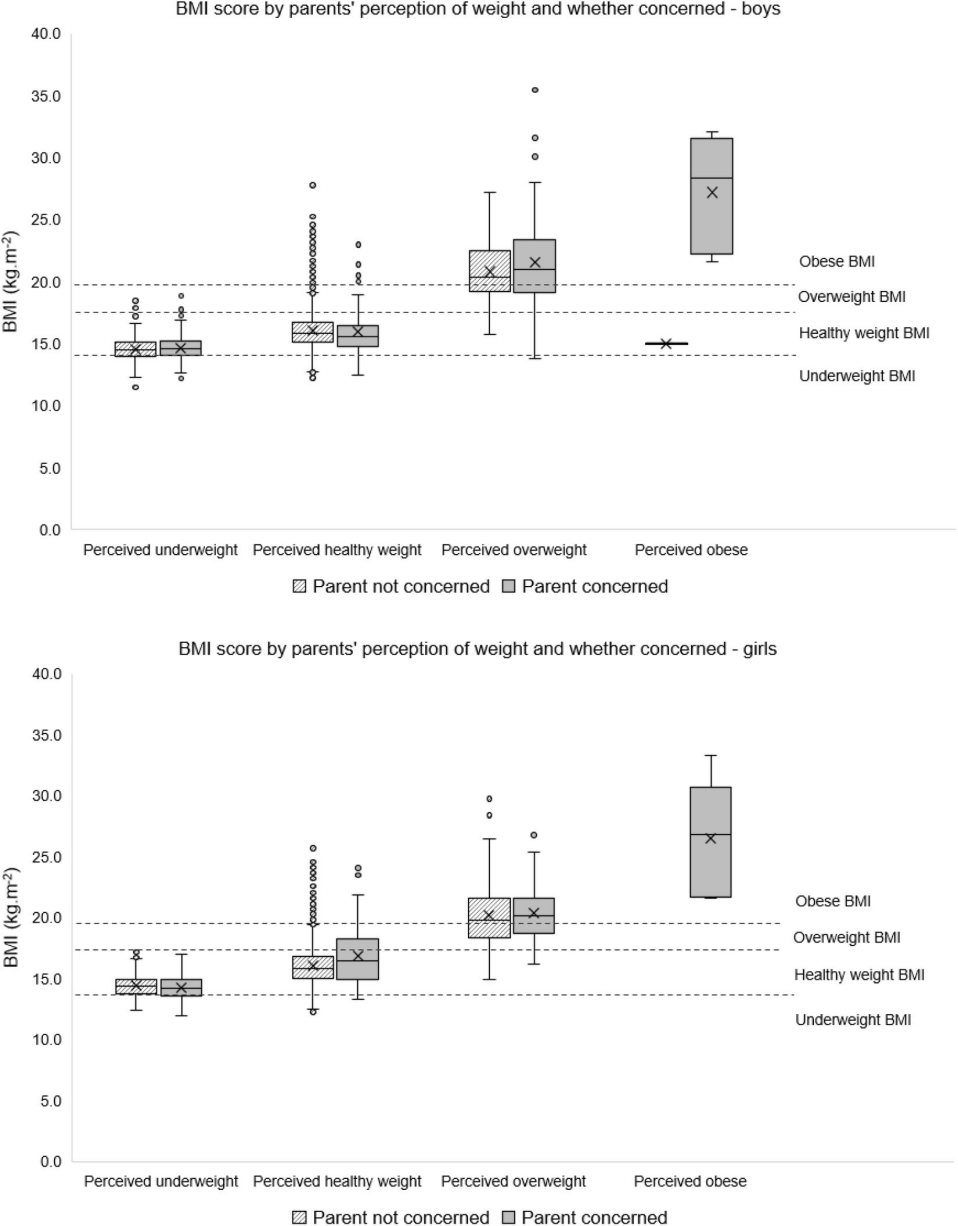
Distribution of BMI score, 2014–2017. Data are presented by parents’ perception of weight and whether they expressed concerns.

Figure 2 shows the distribution of BMI for boys and girls stratified by parents’ perceptions and concerns. For children perceived as underweight or of healthy weight, their BMI was predominately in the healthy BMI range (69.7% of boys perceived as underweight and 82.7% perceived as healthy weight, measured as healthy weight; 67.0% of girls perceived as underweight and 79.2% perceived as healthy weight, measured as healthy weight). For those perceived as overweight or obese, the majority measured in the obese BMI range (67.9% of boys and 59.7% of girls). The long tails in the box plots show that there are many children whose parents perceive them to be of healthy weight, when their measured BMI puts them in the overweight or obese range (12.8% of boys and 17.0% of girls).

For the period 2014–2017, the parents of 1318 (7.0%) children indicated they had concerns about their child’s weight (Table 3). This included 5.5% of children who measured in the overweight range, and 29.4% of children who measured in the obese range. Among children who were in the overweight or obese BMI range, mean BMI was associated with parental concern (*p* < 0.001) and perception (overweight/obese vs healthy weight/underweight; *p* < 0.001) (Table 4a), however sex, Indigenous status, socioeconomic status, and having a regular general practitioner (family physician) were not.

**Table 3:** Reported parental concerns about weight by measured body mass index, and number of comments received.

Measured BMI	Parental concerns about child’s weight			Parental comments provided about child’s weight
	Yes	No	Total	Total
	Number (percent of total BMI)			
	2001–2017			
Underweight	796 (17.6)	3732 (82.4)	4528	n.a.
Healthy weight	2218 (4.2)	49,987 (95.8)	52,205	n.a.
Overweight	371 (4.8)	7345 (95.2)	7716	n.a.
Obese	675 (28.6)	1683 (71.4)	2358	n.a.
*Overweight or obese*	*1046 (10.4)*	*9028 (89.6)*	*10,074*	*n.a*.
Total	4060 (6.1)	62,747 (93.9)	66,807	n.a.

	2014–2017			
Underweight	251 (22.5)	863 (77.5)	1114	234 (21.0)
Healthy weight	743 (5.1)	13,923 (94.9)	14,666	1105 (7.5)
Overweight	126 (5.5)	2146 (94.5)	2272	198 (8.7)
Obese	198 (29.4)	476 (70.6)	674	141 (20.9)
*Overweight or obese*	*324 (11.0)*	*2622 (89.0)*	*2946*	*339 (11.5)*
Total	1318 (7.0)	17,408 (93.0)	18,726	1678 (9.0)

**Table 4:** a. Characteristics of children who measured as being in the overweight and obese BMI range by parental concern and parental perception of weight, 2014–2017. b. Characteristics of children measured as having healthy BMI by parental concern and parental perception of weight, 2014–2017.

Characteristic		Parental concern about weight (*n* = 2946)			Parental perception about weight (*n* = 2868)		
		Yes (*n* = 324)	No (*n* = 2622)		Overweight or obese (*n* = 382)	Healthy weight or underweight (*n* = 2486)	
		Mean (SD)	Mean (SD)	*p* value	Mean (SD)	Mean (SD)	*p* value
Body mass index (kg.m^−^^2^)	Male	21.59 (3.25)	18.8 (1.45)	<0.001	21.63 (3.02)	18.64 (1.23)	<0.001
	Female	20.42 (2.52)	18.59 (1.44)	<0.001	20.8 (2.56)	18.45 (1.21)	<0.001
		Number (%)	Number (%)	*p* value (χ^2^)	Number (%)	Number (%)	*p* value (χ^2^)
Sex	Male	138 (10.6)	1170 (89.4)	0.488	177 (13.9)	1095 (86.1)	0.402
	Female	186 (11.4)	1452 (88.6)		205 (12.8)	1391 (87.2)	
Indigenous status	Aboriginal or Torres Strait Islander	8 (9.5)	76 (90.5)	0.654	9 (11.5)	69 (88.5)	0.631
	Not Aboriginal or Torres Strait Islander	316 (11.1)	2537 (88.9)		373 (13.4)	2408 (86.6)	
Relative socioeconomic disadvantage	Most disadvantaged (Q1)	68 (11.7)	513 (88.3)	0.700	77 (13.9)	477 (86.1)	0.151
	Quintile 2	74 (12.3)	526 (87.7)		95 (16.1)	496 (83.9)	
	Quintile 3	65 (10.8)	535 (89.2)		78 (13.3)	508 (86.7)	
	Quintile 4	56 (9.9)	510 (90.1)		61 (10.9)	497 (89.1)	
	Least disadvantaged (Q5)	53 (10.5)	451 (89.5)		63 (13.0)	423 (87.0)	
Whether has a usual General Practitioner	Yes	267 (10.7)	2229 (89.3)	0.272	317 (13.0)	2115 (87.0)	0.343
	No	49 (12.6)	341 (87.4)		56 (14.8)	322 (85.2)	

Characteristic		Parental concern about weight (*n* = 14,666)			Parental perception about weight (*n* = 14,351)			
		Yes (*n* = 743)	No (*n* = 13,923)		Overweight or obese (*n* = 21)	Healthy weight (*n* = 13,519)	Underweight (*n* = 811)	
		Mean (SD)	Mean (SD)	*p* value	Mean (SD)	Mean (SD)	Mean (SD)	*p* value
Body mass index (kg.m^−2^)	Male	15.18 (0.75)	15.73 (0.84)	<0.001	16.13 (0.72)	15.75 (0.83)	14.99 (0.65)	<0.001
	Female	15.09 (0.89)	15.61 (0.86)	<0.001	16.63 (0.57)	15.63 (0.85)	14.85 (0.74)	<0.001
		Number (%)	Number (%)	*p* value (χ^2^)	Number (%)	Number (%)	Number (%)	*p* value (χ^2^)
Sex	Male	466 (6.0)	7249 (94.0)	<0.001	6 (0.1)	7045 (93.4)	490 (6.5)	<0.001
	Female	277 (4.0)	6674 (96.0)		15 (0.2)	6474 (95.1)	321 (4.7)	
Indigenous status	Aboriginal or Torres Strait Islander	19 (6.0)	300 (94.0)	0.456	0 (0.0)	282 (91.9)	25 (8.1)	n.a.
	Not Aboriginal or Torres Strait Islander	720 (5.0)	13,591 (95.0)		21 (0.1)	13,205 (94.3)	782 (5.6)	
Relative socioeconomic disadvantage	Most disadvantaged (Q1)	147 (5.8)	2375 (94.2)	0.161	1 (0.0)	2295 (93.4)	162 (6.6)	n.a.
	Quintile 2	144 (5.1)	2656 (94.9)		5 (0.2)	2577 (94.4)	149 (5.5)	
	Quintile 3	169 (5.4)	2943 (94.6)		6 (0.2)	2857 (93.9)	181 (5.9)	
	Quintile 4	125 (4.4)	2731 (95.6)		3 (0.1)	2656 (95.1)	135 (4.8)	
	Least disadvantaged (Q5)	138 (5.0)	2637 (95.0)		6 (0.2)	2570 (94.1)	156 (5.7)	
Whether has a usual General Practitioner	Yes	614 (4.9)	11,993 (95.1)	0.043	20 (0.2)	11,662 (94.4)	674 (5.5)	n.a.
	No	111 (6.0)	1750 (94.0)		1 (0.1)	1687 (93.5)	117 (6.5)	

Results were similar for children in the healthy weight BMI range, with the exception of sex. Carers of male children had significantly higher concerns about their child’s weight when compared to carers of female children (6.0% vs. 4.0%, *p* < 0.001) and a higher proportion of male children with a healthy BMI were perceived as being underweight when compared to female children with healthy BMI (6.5% vs. 4.7%, *p* < 0.001) (Table 4b).

### Qualitative analysis

Parental comments were generally explanatory in nature providing further details about why parents were or were not concerned about their child’s height or weight. However common themes differed amongst those children later measured as having BMIs that were underweight, healthy or overweight/obese.

For those children whose BMI was measured in the underweight category parent comments were often simple explanations of known issues that might explain underweight;

*“Had gastrostomy until 2 yrs of age. Hx failure to thrive. Has multiple specialists in Sydney.”*

but also included justifications of why parents had stated they did not have concerns;

*“he is a slight build & quite little, but he eats well, it is not uncommon for Filipinos”*.

Parents of children whose measured BMI was in the healthy range included comments which indicated that parents were actively concerned their child was underweight;

*“I’m trying to put some weight on him.”*

*“Just needs to put on maybe 2–4 kilos to be healthy.”*

Others displayed uncertainty about what was normal or healthy;

*“Probably underweight. Can see one bone protrude-is that normal?”*

There were three common themes found in the comments of parents of children who had a high BMI (overweight or obese category). Many parents described family traits or body type as if for justification or explanation;

*“Big and tall for his age- genetically all family members on dad’s side are built tall/solid build”*.

Some parents provided information about medical issues which would affect BMI;

*“His epilepsy medication can have an effect on his weight. Paediatrician is happy”*.

Others explained actions they were taking to address a concern about height or weight;

*“We have a family history of being overweight. We are very conscious of this; she still has trouble in this area & would love some help with this”*

*“We had some concerns about her weight-addressing through exercise & healthy choices”*.

Parents of children whose measured BMI was in the overweight/obese category also wrote comments which displayed an awareness of, but scepticism about BMI as an indicator that could/would apply to their child;

*“Is very tall for her age and eats accordingly. I don’t like that her BMI states she is obese and I feel that her height and weight are in comparison”*

*“Very active, eats healthily, short and solid. BMI is not a true indicator in this instance.”*

These comments were not present from parents with children with healthy or low BMI.

## Discussion

### Main findings

We found parents frequently perceived their child to be lighter than their measured BMI, where the child was in the overweight or obese BMI range. The vast majority parents of children with overweight or obesity perceived their child as being of healthy weight (86.4%) and over one in twenty parents (5.7%) of children with healthy weight perceived their child as underweight. Over one in seven (15.8%) ACT Kindergarten children measured as overweight or obese in 2014–2017, with data from 2001 showing the proportion of children measuring as overweight or obese being relatively stable over the years. Fewer than half of parents of children with overweight or obesity indicated having concerns about their child’s weight, and comments from these parents varied from documenting their concern and response, to active rejection of BMI as relevant or timely.

Average BMI was fairly steady over the period 2001–2017, with a range of 0.30 kg.m^2^ for both boys and girls (equivalent to 830 g in a boy of average height and 865 g for a girl of average height). While these BMI fluctuations are small, if all boys and girls measuring as overweight or obese weighed 850 g less, this would equate to nearly 4000 (5.5%) more ACT Kindergarten children being classified as healthy weight during 2001–2017. Worldwide, average BMI is continuing to increase, although a levelling out of trends has been seen in high-income English-speaking countries for children and adolescents aged 5–19 years [21], which is consistent with the stable results that we observed in our high average socioeconomic status population. Several hypotheses have been proposed to explain this stabilisation, including the implementation of successful interventions, and reaching maximal environmental ‘obesenogenicity’ [22]. Interventions are often driven by government policy. The Australian government set targets for increasing the proportion of children at healthy body weight in 2008, and rates of overweight and obesity remain key Children’s Headline Indicators [23, 24]. The proportion of children aged 5–14 years who measured as overweight or obese increased from 23.1% in 2007–08 to 26.0% in 2011–12, but remained stable at 26.1% in 2014–15 [24]. Within the ACT, the territory government released their *Healthy Weight Action Plan* in 2013, with a goal of zero increase in rates of overweight and obesity, and interventions to improve physical health for children and adults alike focussing on nutrition and physical activity [25]. Local data suggest that the proportion of children aged 5–17 years in the ACT classified as overweight or obese has decreased in 2015–2016 to 20.6% from 26.3% in 2013–14, however it is noted that the decrease was not statistically significant [26].

We found that among children with overweight or obesity in 2014–2017, the majority their parents perceived their child to be of healthy weight, with only 13.3% identifying their child as being either overweight or obese, and only 11.0% indicating concern about their child’s weight. Our results are broadly consistent with other studies showing a mismatch between how parents perceive their child’s weight and objective weight measures [14]. Studies from Portugal, the Netherlands and New Zealand have demonstrated similar disparities for young children; 17% of overweight Portuguese 2–6 year-olds, 15% of overweight Dutch 5-year-olds, and 42% of overweight 2–8 year-old New Zealanders, were perceived as overweight by their parents [27–29].

Disparities between parental concerns and child weight are not new in the Australian setting. Analysis of the 2004 cohort of the Longitudinal Survey of Australian Children found 23% of parents of children aged 4–5 years with overweight or obesity perceived them as such [30]. A 2001 sample of 5–6-year-old children and their parents from Melbourne, Australia, found that just 11% of parents saw their child with overweight as such [31]. While only 29% of parents of children with overweight in that study were concerned about their child’s weight at that time, 46% expressed concerns that they would be overweight as an adolescent. A study of children aged 5–13 years in 1997–99 from Victoria, Australia reported 81% of parents of children with overweight and 42% of parents of children with obesity did not report concern about their child’s weight [32]. Concern was associated with the child’s BMI, but not with their sex, or parents’ BMI or education.

In our study, both parental perceptions and concerns were associated with their child’s measured BMI, but there was no association observed with sex, Indigenous status, socioeconomic status, or having a regular general practitioner—despite Aboriginal and Torres Strait Islander children, and those from relatively disadvantaged socioeconomic groups having higher rates of overweight and obesity. Literature reviews have reported mixed results on factors relating to underestimation of child weight in overweight children [33, 34]. Many studies found no association between factors such as sex, ethnicity, child age, parental weight and parent education level and misperception, although there were some indicating that parents of boys, younger children, and those of lower educational level were more likely to be inaccurate in their perception [35]. Interestingly, parents of girls and those of older children are more likely to express concerns about their child’s weight [10, 31, 34]. Studies of factors associated with misperception and concern are heterogeneous, so it is difficult to make broad inference. One factor affecting parental assessment of children’s weight is thought to be the changing distribution of weight around the world, with overweight and obesity becoming normalised as their prevalence increases [3, 35]. This was seen in the qualitative responses provided by parents in our study, where some noted that their child’s build was consistent with others in their family. A review by Robinson [35] collated evidence from numerous studies supporting the idea of “visual recalibration”, where what is seen as ‘normal’ changes as overweight and obesity become more common, and there is increased underestimation of body weight status—by parents and health professionals alike.

Our study showed poor correlation between children’s measured weight status and their parents’ perception of their weight, and low levels of parental concern about their children’s weight, even among those who were classified as overweight or obese. These parents could be classified as “pre-contemplative” in the transtheoretical model of stages of change [36]. Health promotion messages directed at reducing childhood obesity are unlikely to be effective if parents do not perceive them to be relevant to them and their children. While a plethora of research has shown that parents who are more aware of their child’s weight status are more likely to encourage healthy behaviours in their children, other studies have raised doubts about the utility of correct parental perception and concern in children with overweight or obesity. Research has found that children perceived as overweight by their parents have greater relative weight gain [27, 30, 37, 38]. Children perceived as overweight were seen to have engaged in more dieting for weight loss, and concerns about a child’s weight may lead to restrictive or controlling parenting rather than engaging in health-promoting practices [10, 30, 39]. None of these studies imply a causal relationship between perceptions and weight gain, however they do suggest that parental awareness alone is not sufficient to improve the health of children, and that parents themselves need support to achieve this. In this study parental comments indicated some scepticism or concern about BMI as an indicator.

Several studies have assessed the relationship between various factors and parents’ perceptions and concerns about their child’s weight, but to our knowledge this is the first to consider the reasons parents themselves present. Analysis of parents’ comments of children who are measured in the overweight or obese category reveals comments from the whole spectrum of behaviour change model. Many parents’ comments show they are pre-contemplative; either unaware their child has a potential health issue, justifying it or actively denying the role of BMI as a useful measure. Others comments are contemplative—unsure if there is a problem and seeking help or clarification. Much fewer comments reflect parents in the active or maintenance phase in that they are aware of the issue and managing it. Not all parents provided free-text comments but the quantitative analysis with a large proportion of parents who perceive their children’s measured weight inaccurately implies a need for shift in perception and knowledge before more behaviour change is likely.

Previous studies indicated that parents had difficulty defining overweight in childhood and commonly just compared their child with others [40]. Thus there is a conflation between ‘normal/average’ weight and healthy weight which, as the average weight of child and adult populations increases, makes correct identification of health risk more challenging for parents.

On analysis of trends it was observed that the 2005 data were inconsistent with other years. Data were investigated for each school, and it was found that the inconsistent results clustered in schools. This was investigated and thought likely due to an error in calibration of one set of equipment.

### Strengths and limitations

The KHC is a long-running complete enumeration survey with excellent response rates, giving a large representative sample for analysis. BMI was derived from measured height and weight, which is known to be more accurate than self-reported data [41].

As the KHC is a series of cross-sectional surveys we can demonstrate relationships between variables, but not causality. Other studies have found, for example, that mothers reporting concern about their child’s weight reported greater influence on their child’s diet and physical activity, however the temporal relationship could not be determined [42]. The impact of this influence is unknown, as discussed by Rodgers et al. [43]. who note that controlling a child’s diet is not the same as reducing how the obesogenic the environment is.

Our study is unique as we have access to free-text comments from parents about their child’s weight, which we can assess against their reported perceptions and concerns, as well as measured height and weight data. Qualitative analysis was done on free-text comments that were provided by fewer than half of the parents and are illustrative rather than generalisable.

A limitation of the data is that the parents’ comments are typically collected at a different point in time from when children have their height and weight measured. Physical assessments took place an average of 5.2 months (SD 2.18; median 5 months) from the completion of the questionnaire. It is therefore possible that for some children, their BMI has changed during this timeframe.

A potential limitation of using an area-based measure to create quintiles of socioeconomic disadvantage is that this reflects the area children live in rather than their personal attributes. However as geocoded data were available, the areas used were the finest available.

We omitted missing data from our analysis, without making adjustments, thereby effectively using implicit imputation. If the characteristics of children who did not participate in all or part of the study are different from those who did fully participate, and if these characteristics in turn relate to our variables of interest, there is potential to introduce bias into the results.

## Conclusion

Our study found that parents do not accurately perceive their child’s weight, which is consistent with results seen worldwide. We also found very low levels of parental concern for their child’s weight, even among children with obesity. This suggests that parents may need external input to inform them about their child’s health status. This can be achieved through health checks such as the KHC, or in routine review with their general practitioner. Parental awareness of child overweight or obesity is not sufficient however, and education and support are needed to help achieve improve health for young children and to navigate the obesogenic environment in a positive way.

## Supplementary information

Kindergarten Health Check - Consent and Questionnaire 2017

STROBE checklist
