# Supplementary material for: Parents’ perceptions of their child’s weight among children in their first year of primary school: a mixed-methods analysis of an Australian cross-sectional (complete enumeration) study
Source: Int J Obes (Lond). 2022 Jan 24;46(5):992–1001. doi: 10.1038/s41366-022-01068-5 (PMC9050591; doi:10.1038/s41366-022-01068-5)
Supplement: Supplementary file 1 — Kindergarten Health Check - Consent and Questionnaire 2017 [file 41366_2022_1068_MOESM1_ESM.pdf]

* 3 7 6 2 5 *

ACT Health

Kindergarten Health Check Consent and Questionnaire 2017

URN : ______ *Office use only*

Please complete all details below in black pen

PERSONAL DETAILS OF YOUR CHILD

Family name: ____________________

Given names: ____________________

DOB: ____/____/____ **Gender:** __________

School: ____________________ Roll group / Class: ____________________

Home Address: ____________________

Suburb / Town: ____________________ Postcode: ____________________

Postal Address (if different to home address): ____________________

Suburb / Town: ____________________ Postcode: ____________________

Is your child of Aboriginal or Torres Strait Islander origin?

☐ No ☐ Yes, Aboriginal

☐ Yes, Torres Strait Islander ☐ Yes, both Aboriginal and Torres Strait Islander

Does your child have a General Practitioner (GP) or Practice? ☐ Yes ☐ No

Name of Family Doctor: ____________________ Practice Name: ____________________

Address: ____________________

Suburb: ____________________ Postcode: __________ Phone: ____________________

CONSENT FOR HEALTH CHECK

I/we have read and understood the Kindergarten Health Check information and I/we consent to the health check as described on the information sheet. ☐ Yes ☐ No

I/we consent to the School Health Nurse alerting my child's teacher to concerns relating to hearing and vision only. ☐ Yes ☐ No

Signature/s: ____________________
Parent / Legal Guardian

Print name/s: ____________________ **Date:** ____/____/2017
(Please print clearly in BLOCK LETTERS)

Parent / Guardian Contact Details

In listing your contact details you are giving the School Health Nurse permission to contact you regarding the Kindergarten Health Check, if required.

Home phone: ____________________

Best day time contact number: ____________________

Mobile/s: ____________________

____

Kindergarten Health Check

These questions ask you about your child's vision and hearing. Please tick ✓ the required boxes.

VISION

1. Do you have any concerns about your child's vision? ☐ Yes ☐ No

   If yes, please describe:____

2. Has your child been prescribed glasses? ☐ Yes ☐ No

   If yes, when should they be worn? (e.g. when reading):________________________

3. Has your child ever received, or are they receiving medical care for their eyes or vision? ☐ Yes ☐ No

   If yes, please describe:____

HEARING

1. Do you have any concerns about your child's hearing or airways? ☐ Yes ☐ No

   If yes, please describe:____

2. Has your child had any of the following? Tick all that apply.

   Repeated ear infections ☐ Yes ☐ No

   Discharging ears ☐ Yes ☐ No

   Hearing Loss ☐ Yes ☐ No

   Grommets ☐ Yes ☐ No If yes, when were these inserted? ____________________

   Snoring ☐ Yes ☐ No

3. Has your child ever received or are they receiving medical care for their ears, hearing or airways?

   ☐ Yes ☐ No

   If yes, please describe:____

Would you like information on any of the following? Tick all that apply.

Wetting pants ☐ Yes ☐ No

Wetting the bed ☐ Yes ☐ No

Soiling pants ☐ Yes ☐ No

This completes the questions relating to the health check conducted by the School Health Nurses.

The results will be posted to your nominated address.

Please continue answering the questions about your child's development on the following pages.

If you have any concerns about your child's health, please see your GP.

* 3 7 6 2 5 *

ACT Health

Kindergarten Health Questionnaire

URN : ☐☐☐☐☐☐☐☐ *Office use only*

Please complete all details below in black pen

PERSONAL DETAILS OF YOUR CHILD

Family name: ______________________________

Given names: ______________________________

DOB: **Gender:**

These questions ask you about your child's development.

PARENTS' EVALUATION OF DEVELOPMENTAL STATUS (PEDS)

1. Please list any concerns about your child's learning, development and behaviour.

2. Do you have any concerns about how your child talks and makes speech sounds?
   ☐ No ☐ Yes ☐ A little Comments:

3. Do you have any concerns about how your child understands what you say?
   ☐ No ☐ Yes ☐ A little Comments:

4. Do you have any concerns about how your child uses his or her hands or fingers to do things?
   ☐ No ☐ Yes ☐ A little Comments:

5. Do you have any concerns about how your child uses his or her arms or legs?
   ☐ No ☐ Yes ☐ A little Comments:

6. Do you have any concerns about how your child behaves?
   ☐ No ☐ Yes ☐ A little Comments:

7. Do you have any concerns about how your child gets along with others?
   ☐ No ☐ Yes ☐ A little Comments:

8. Do you have any concerns about how your child is learning to do things for himself / herself?
   ☐ No ☐ Yes ☐ A little Comments:

9. Do you have any concerns about how your child is learning preschool or school skills?
   ☐ No ☐ Yes ☐ A little Comments:

10. Please list any other concerns..

© Authorised Australian Version, The Royal Children's Hospital, Centre for Community Child Health. Adapted with permission from Frances Page Glascoe and PEDSTest.com LLC.

These questions ask you about asthma, eczema and hay fever.

RESPIRATORY SYMPTOMS

1. Has your child ever had wheezing or whistling in the chest? ☐ Yes ☐ No

2. Has your child ever had asthma? ☐ Yes ☐ No

3. In the last 12 months has your child experienced any of the following respiratory symptoms?

   Wheezing or whistling in the chest ☐ Yes ☐ No

   A dry cough at night not associated with a cold or chest infection ☐ Yes ☐ No

   Wheezing with coughs or colds ☐ Yes ☐ No

   Shortness of breath when exercising or playing games or participating in sports ☐ Yes ☐ No

4. In the last 12 months how often, *on average*, have the following respiratory symptoms been present?
   Please tick one box on EACH line

Wheeze or whistle in the chest	☐ Never	☐ Less than 1 day / wk	☐ 1 – 3 days / wk	☐ 4 or more days / wk
Night cough or night wheeze	☐ Never	☐ Less than 1 night / wk	☐ 1 – 3 nights / wk	☐ 4 or more nights / wk
Shortness of breath (when exercising or playing)	☐ Never	☐ Less than 1 day / wk	☐ 1 – 3 days / wk	☐ 4 or more days / wk

ECZEMA

5. Has your child ever had an itchy rash that was coming and going for at least 6 months? ☐ Yes ☐ No

6. Has the itchy rash ever affected the following places? ☐ Yes ☐ No
(the fold of the elbows, behind the knees, in front of the ankles, under the buttocks, or around the neck, ears or eyes)

7. Has your child ever had eczema? ☐ Yes ☐ No

HAY FEVER

8. In the past 12 months has your child had a problem with sneezing, or a runny or blocked nose when he / she did not have a cold or the flu? ☐ Yes ☐ No

9. In the past 12 months has this nose problem been accompanied by itchy / watery eyes? ☐ Yes ☐ No

10. Has your child ever had hay fever? ☐ Yes ☐ No

11. Do any close members of the family have any of the following conditions?

    Asthma ☐ Yes ☐ No

    Eczema ☐ Yes ☐ No

    Hay fever ☐ Yes ☐ No

Adapted from The International Study of Asthma and Allergies in Childhood (ISAAC): Core questionnaire

These questions ask about your child's food intake and physical activity.

WEIGHT PERCEPTION

1. How would you describe your child's weight?

   ☐ Underweight

   ☐ Healthy weight

   ☐ Overweight

   ☐ Obese

   ☐ Don't know

2. Do you have any concerns about your child's weight? ☐ Yes ☐ No

3. Do you have any concerns about your child's height? ☐ Yes ☐ No

Comments: ____

DIET

The following questions are about the food your child eats.

4. How many serves of vegetables does your child usually eat each day?
(1 serve = ½ cup cooked vegetables, or ½ medium potato, or 1 medium tomato, or 1 cup salad vegetables)

   ___ serves per day *(write number of serves) OR*

   ___ serves per week *(write number of serves)*

   ☐ my child doesn't eat vegetables

   ☐ don't know

5. How many serves of fruit does your child usually eat each day?
(1 serve = 1 medium piece, or 2 small pieces of fruit, or 1 cup of diced pieces with no added sugar, or 30 grams of dried fruit such as 4 dried apricots or 1½ tablespoons sultanas)

   ___ serves per day *(write number of serves) OR*

   ___ serves per week *(write number of serves)*

   ☐ my child doesn't eat fruit

   ☐ don't know

6. Do you have any concerns about your child's eating habits? ☐ Yes ☐ No

Comments: ____

PHYSICAL ACTIVITY

The following questions are about your child's physical activity. Physical activity is any activity that increases your heart rate and makes you get out of breath some of the time.

7. On about how many days <u>during the school week</u> does your child usually do physical activity outside of school hours? This includes before and after school sports, playing with friends, walking/ riding to and from school.

   ___ number of school days per week
   ☐ none
   ☐ don't know

8. On these days, about how many hours does your child usually do physical activity? *(in hours and minutes)*

   ___ hours ___ minutes (average time on each school day)
   ☐ don't know

9. On about how many days <u>during the weekend</u> does your child usually do physical activity?

   ___ number of weekend days
   ☐ none
   ☐ don't know

10. On a typical weekend day, about how many hours does your child usually do physical activity?

   ___ hours ___ minutes (average time on each weekend day)
   ☐ don't know

Strengths and Difficulties Questionnaire

P or T 4-10

For each item, please mark the box for Not True, Somewhat True or Certainly True. It would help us if you answered all items as best you can even if you are not absolutely certain. Please give your answers on the basis of the child's behaviour over the last six months or this school year.

Child's name .... Male/Female

Date of birth..

	Not True	Somewhat True	Certainly True
Considerate of other people's feelings	☐	☐	☐
Restless, overactive, cannot stay still for long	☐	☐	☐
Often complains of headaches, stomach-aches or sickness	☐	☐	☐
Shares readily with other children, for example toys, treats, pencils	☐	☐	☐
Often loses temper	☐	☐	☐
Rather solitary, prefers to play alone	☐	☐	☐
Generally well behaved, usually does what adults request	☐	☐	☐
Many worries or often seems worried	☐	☐	☐
Helpful if someone is hurt, upset or feeling ill	☐	☐	☐
Constantly fidgeting or squirming	☐	☐	☐
Has at least one good friend	☐	☐	☐
Often fights with other children or bullies them	☐	☐	☐
Often unhappy, depressed or tearful	☐	☐	☐
Generally liked by other children	☐	☐	☐
Easily distracted, concentration wanders	☐	☐	☐
Nervous or clingy in new situations, easily loses confidence	☐	☐	☐
Kind to younger children	☐	☐	☐
Often lies or cheats	☐	☐	☐
Picked on or bullied by other children	☐	☐	☐
Often volunteers to help others (parents, teachers, other children)	☐	☐	☐
Thinks things out before acting	☐	☐	☐
Steals from home, school or elsewhere	☐	☐	☐
Gets along better with adults than with other children	☐	☐	☐
Many fears, easily scared	☐	☐	☐
Good attention span, sees work through to the end	☐	☐	☐

Signature .... Date ....

Parent / Teacher / Other (Please specify):

Thank you very much for your help

© Robert Goodman, 2005
